# A case of VEXAS syndrome (vacuoles, E1 enzyme, X-linked, autoinflammatory, somatic) with decreased oxidative stress levels after oral prednisone and tocilizumab treatment

**DOI:** 10.3389/fmed.2022.1046820

**Published:** 2022-12-05

**Authors:** Nagie Tozaki, Chisato Tawada, Hirofumi Niwa, Yoko Mizutani, En Shu, Aki Kawase, Yuki Miwa, Hidenori Ohnishi, Hideo Sasai, Keisuke Miyako, Junichi Hosokawa, Ayaka Kato, Kazuhiro Kobayashi, Tatsuhiko Miyazaki, Yohei Shirakami, Masahito Shimizu, Hiroaki Iwata

**Affiliations:** ^1^Department of Dermatology, Gifu University Graduate School of Medicine, Gifu, Japan; ^2^Department of Pediatrics, Gifu University Graduate School of Medicine, Gifu, Japan; ^3^Department of Applied Genomics, Kazusa DNA Research Institute, Chiba, Japan; ^4^Department of General Medicine, Gifu University Graduate School of Medicine, Gifu, Japan; ^5^Department of Pathology, Gifu University Graduate School of Medicine, Gifu, Japan; ^6^Department of Gastroenterology, Gifu University Graduate School of Medicine, Gifu, Japan

**Keywords:** VEXAS, vacuoles, E1 enzyme, X-linked, autoinflammatory diseases, somatic, tocilizumab

## Abstract

VEXAS (vacuoles, E1 enzyme, X-linked, autoinflammatory, somatic) syndrome has recently been described as an autoinflammatory disease associated with severe adult-onset inflammatory manifestations. The various clinical manifestations include recurrent high-grade fever, neutrophilic dermatoses, cutaneous vasculitis, chondritis of the ear and nose, pulmonary infiltrates, cytopenia, uveitis, gastrointestinal pain or inflammation, aortitis, hepatosplenomegaly, and hematological disorders. VEXAS syndrome is caused by somatic mutations of the ubiquitin-like modifier activating enzyme 1 (*UBA1*) gene in myeloid-lineage cells. It is characterized by vacuolated myeloid and erythroid progenitor cells seen by bone marrow biopsy. We report the case of a 64-year-old Japanese man with VEXAS syndrome. At age 63, he was referred to us with a recurrent erythema on the hands associated with a general fever of 38–40°C that had persisted for 4 or 5 days and had recurred about once a month for a year. The skin rash appeared 2 or 3 days after the onset of each fever episode. Computed tomography (CT) of the chest revealed bilateral hilar lymphadenopathy (BHL), and the mediastinal lymph nodes were swollen. Sarcoidosis was suspected but was ruled out by several tests. Laboratory examinations showed elevated inflammatory markers. Bone marrow examination showed the vacuolization of myeloid precursor cells. A skin biopsy revealed dense dermal, predominantly perivascular, infiltrates. These consisted of mature neutrophils admixed with myeloperoxidase-positive CD163-positive myeloid cells, lymphoid cells and eosinophils. Sequencing analysis identified the somatic UBA1 variant c.122T > C, which results in p.Met41Thr. Treatment with oral prednisone (15 mg/day) and monthly intravenous tocilizumab injections (400 mg) completely resolved the symptoms. Neutrophils are a major source of reactive oxygen species, and the present case demonstrated numerous neutrophilic infiltrates. We hypothesize that the patient might have had elevated derivatives of reactive oxygen metabolites (d-ROMs). d-ROM quantification is a simple method for detecting hydroperoxide levels, and clinical trials have proven it useful for evaluating oxidative stress. In this study, we measured serum d-ROM before and after oral prednisone and tocilizumab treatment. The levels decreased significantly during treatment.

## Introduction

VEXAS (vacuoles, E1 enzyme, X-linked, autoinflammatory, somatic) syndrome is the first example of an autoinflammatory disease arising exclusively from somatic mutations and is one of an emerging class of acquired errors of immunity. Although this disorder was only first reported in 2020, hundreds of patients have been identified (1–5). VEXAS syndrome is caused by pathogenic variants in ubiquitin-like modifier activating enzyme 1 (*UBA1*), a gene on the X chromosome that encodes UBA1, and these mutations are restricted to myeloid-lineage cells (1). Patients with this syndrome are characterized by adult-onset recurrent fever, neutrophilic dermatoses, leukocytoclastic vasculitis, polyarteritis nodosa, ear and nose chondritis, venous thromboembolism, pulmonary infiltrates, gastrointestinal pain or inflammation, aortitis, hepatosplenomegaly, macrocytic anemia, elevated acute-phase reactants, myelodysplastic syndrome (MDS), multiple myeloma, and bone marrow vacuolization restricted to myeloid and erythroid precursor cells (1–5). The majority of the patients have myeloid lineage-restricted somatic mutations in *UBA1*, affecting the Met41 residue of the protein. These mutations promote the production of an inactive isoform (UBA1c) from a downstream translation site (Met67), resulting in hyperinflammation and decreased ubiquitylation (1, 5).

Autoinflammatory diseases are characterized by neutrophil activation. Neutrophils are a major source of reactive oxidative species (ROS) *via* NADPH oxidase. It is difficult to measure ROS directly due to their quick metabolization. However, derivatives of reactive oxygen metabolites (d-ROMs) are relatively easy to measure, and they reflect the hydroperoxide levels. We describe the clinical course of a VEXAS syndrome patient with a somatic *UBA1* variant who was treated with an oral steroid and anti-IL-6 therapy (tocilizumab) that lead to the resolution of the systemic symptoms. Interestingly, the elevated d-ROMs decreased significantly after these systemic therapies.

## Case description

**FIGURE 1:**
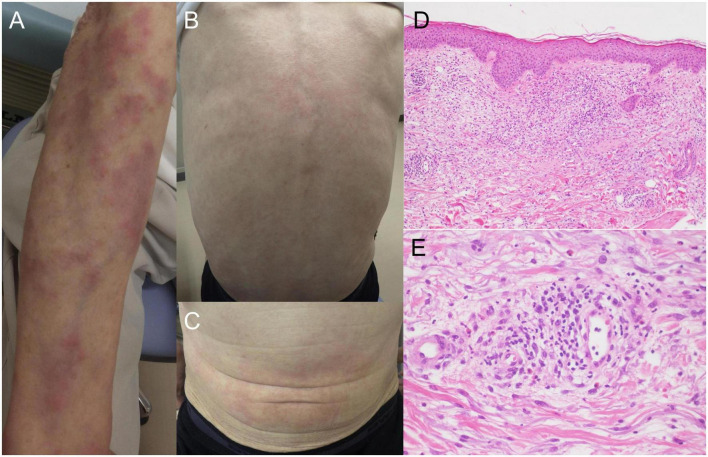
Clinical manifestations and histopathology. **(A)** Multiple erythematous plaques and nodular lesion on the upper limbs. **(B)** Urticarial lesions on the back. **(C)** Erythematous lesions on the abdomen. **(D)** Infiltration of inflammatory cells from the upper dermis to the subcutaneous fat (hematoxylin-eosin stain, original magnification × 100). **(E)** High magnification of dermal infiltrates around the perivascular region. The inflammatory infiltrates contain neutrophils, histiocytes, lymphoid cells, and eosinophils (hematoxylin-eosin stain, original magnification × 400).

**FIGURE 2:**
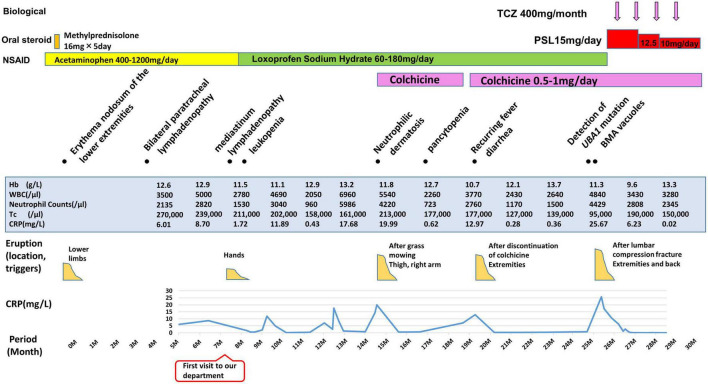
Overview of patient’s disease course. TCZ, tocilizumab; PSL, prednisolone; NSAID, Non-Steroidal Anti-Inflammatory Drug; BMA, bone marrow aspirate; Hb, hemoglobin (normal range, 13.7–16.8 g/L); WBC, white blood cells (normal range, 3,300–8,600/μL); Tc, thrombocytes (normal range, 158,000–348,000/μL); CRP, C-reactive protein (normal < 3 mg/L).

**FIGURE 3:**
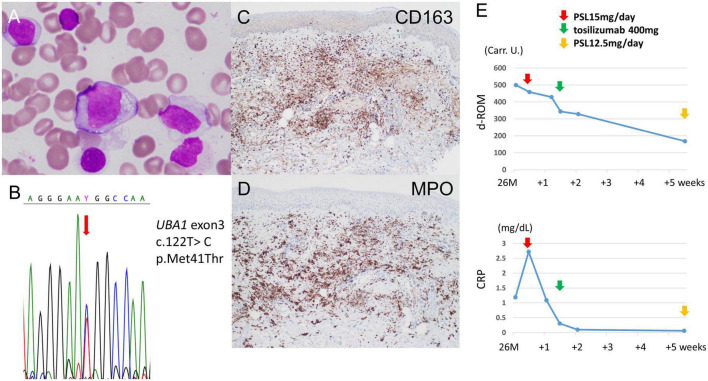
Additional tests for diagnosis and treatment. **(A)** Bone marrow aspirate smears show cytoplasmic vacuolization in myeloblasts (×40 magnification). **(B)** Sanger sequencing of genomic DNA derived from whole peripheral blood. The c.122T > C mutation is detected at position p.Met41 of the *UBA1* gene. **(C)** There are many CD163-positive cells, suggesting macrophages and histiocytes in the dermis (CD163 immunostaining, original magnification × 100). **(D)** The majority of infiltrates are MPO-positive (MPO immunostaining, original magnification × 100). **(E)** Serum d-ROM level before and after treatment. The d-ROM levels are expressed in Carratelli units (Carr. U.). The d-ROM levels decreased significantly after treatment with oral prednisone (15 mg/d) and the first intravenous injections of tocilizumab (400 mg).

**FIGURE 4:**
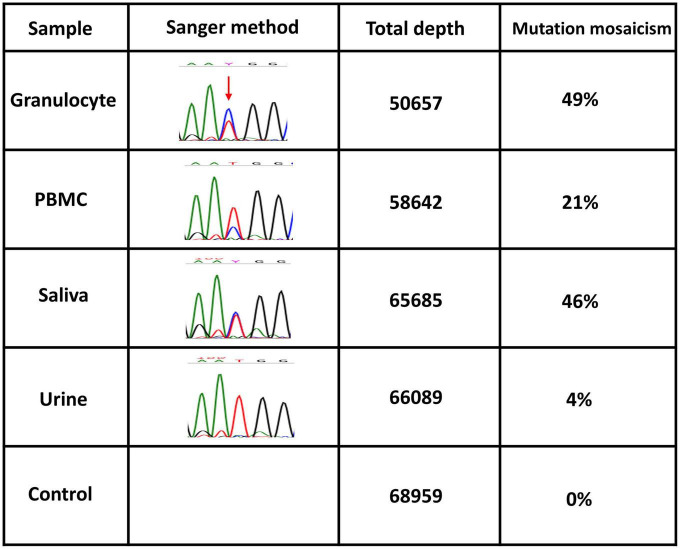
Quantification of mosaicism. *UBA1* somatic variants of granulocytes from peripheral blood, peripheral blood mononuclear cells (PBMCs), saliva, and urine. Mutation mosaicism was analyzed by using the MiSeq Sequencing System (Illumina, Inc.) after polymerase chain reaction (PCR) amplification of the target. The target is the *UBA1* (NM_003334.4) missense mutation c.122T > C (p.Met41Thr) on chromosome X (47199052 T/C). Total depth and mutation mosaicism were measured by bam-readcount (ver 0.8.0). A commercial genome was used as a control.

A 64-year-old Japanese man had a history of surgery for aspergilloma of the lungs at age 60. The patient works for a construction company. His father had died of pneumonia at age 59 and had been suspected of having leukemia. From age 62, he had had fevers of 38–40°C that had recurred about once a month, persisting for 4 or 5 days each time. At the previous hospital, sarcoidosis was suspected due to the swelling of the hilar and mediastinal lymph nodes and erythema nodosum-like cutaneous manifestations. He had been given 16 mg of methylprednisolone for 5 days at his previous hospital based on the diagnosis of erythema nodosum of the lower extremities. At age 63, he was referred to our dermatology department with the chief complaint of erythema on the hands. He was given colchicine for suspected autoinflammatory disease and immunodeficiency. After he started taking oral colchicine, the frequency of his fever decreased to once every 2 or 3 months. However, when the colchicine was stopped, the fever and skin rash flared up. Therefore, the colchicine was resumed and continued. The skin rash repeatedly appeared 2 or 3 days after the onset of fever. At the age of 64 years and 10 months, the fever and skin rash flared up. Erythematous nodules and large and small plaques were seen on the upper extremities (Figure 1A), urticarial papules and plaques on the back (Figure 1B), and diffuse erythema on the abdomen (Figure 1C). A timeline of the clinical manifestations and treatments for more than 2 years is shown in Figure 2. Computed tomography (CT) of the chest revealed bilateral hilar lymphadenopathy (BHL), and the mediastinal lymph nodes were swollen. These were worse than at the previous hospital. Laboratory examinations showed elevated inflammatory markers. The C-reactive protein (CRP) level was elevated to 25.67 mg/dL (normal < 0.14 mg/dL), serum amyloid A protein (SAA) to 1480.0 μg/mL (normal < 8.0 mg/dL), and ferritin to 1034.0 ng/mL (reference range 39.9–465 ng/mL). Hemoglobin was 11.3 g/dL, hematocrit was 32.7%, total white blood cell count (WBC) was 4,840 cells/μL with a normal differential count, and the thrombocyte count was 95,000/μL. Serum protein electrophoresis (SPEP) was negative for M-protein. Rheumatoid factor, antinuclear antibodies, anti-neutrophil cytoplasmic antibodies, myeloperoxidase–anti-neutrophil cytoplasmic antibody (MPO-ANCA), and proteinase3-ANCA (PR3-ANCA) and cryoglobulins were negative. Thymus and activation-regulated chemokine (TARC) was elevated to 944 pg/mL (normal < 450 pg/mL), and IgE was elevated to 1315.0 IU/mL (normal < 232 IU/mL). A polymerase chain reaction (PCR) test was negative for COVID-19 during hospitalization, and the patient had no history of COVID-19 vaccination. Bone marrow examination showed the vacuolization of myeloid precursor cells (Figure 3A). We were able to confirm vacuolization in myeloblast, promyelocyte, myelocyte, and stab cell. A skin biopsy revealed perivascular inflammatory infiltrates in the entire dermis and in the subcutaneous fat (Figure 1D). The infiltrates consisted of mature neutrophils admixed with MPO-positive CD163-positive myeloid cells (Figures 3C,D), lymphoid cells, and eosinophils (Figure 1E). Sequencing analysis of whole peripheral blood identified the *UBA1* variant c.122T > C, which results in p.Met41Thr (Figure 3B). A detailed analysis of mutation mosaicism using the MiSeq Sequencing System (Illumina, Inc.) after the PCR amplification of *UBA1* showed that the variant was found in 49% of granulocytes, 21% of peripheral blood mononuclear cells (PBMCs), 46% of saliva, and 4% of urine (Figure 4). The differential included erythema nodosum, sarcoidosis, adult-onset Still’s disease, and Sweet’s disease. Although the patient met the diagnostic criteria for adult-onset Still’s disease, we made the final diagnosis of VEXAS syndrome based on the clinical manifestations, laboratory tests, histological examinations, and genetic analyses. Treatment with oral prednisone (15 mg/day) was initiated and monthly intravenous injections of tocilizumab (400 mg) achieved the complete resolution of symptoms. At 3 months of follow-up, the patient was clinically stable with gradual improvements in his symptoms. He is currently taking 10 mg/day of prednisolone and we plan to taper the dose.

The d-ROM test measures the amount of hydroperoxide (R-OOH), which is a metabolite produced by active oxygen species and free radicals, in the sample *via* the colorimetric change of a chromogen. The d-ROM value is represented by the arbitrary unit “U.CARR.” The oxidative stress that corresponds to each range of d-ROM values is as follows: normal, 200–300 U.CARR; borderline, 301-320 U.CARR; mild, 321–340 U.CARR; moderate, 341–400 U.CARR; high, 401–500 U.CARR; severe, ≥ 501 U.CARR. A commercial kit was used that involved the colorimetric determination of ROS (d-ROM) using the Free Radical Electron Evaluator (FREE; Health and Diagnostics, Naples, Italy). We measured serum d-ROM levels before and after the treatments in the preset patient. We used a commercial kit and 20 μl of patient serum, which was easy to measure. The d-ROM level was 500 U.CARR before treatment, and it decreased to 429 U.CARR with 4 days of 15 mg/day of oral prednisone. Four days after the intravenous injection of tocilizumab (400 mg), the level had decreased to 329 U.CARR. One month after intravenous tocilizumab, it had decreased to 168 U.CARR. The d-ROM level was significantly decreased after treatment with oral prednisone (15 mg/d) and the first intravenous injection of tocilizumab (400 mg) (Figure 3E). As shown in Figure 2, both CRP and neutrophil counts were elevated when VEXAS syndrome was active. The neutrophil count decreased from 4,429/μL before treatment to 2,808/μL 2 months after the start of treatment.

## Discussion

Systemic corticosteroids and supportive care are the first-line treatment for the inflammatory symptoms and cytopenia of VEXAS syndrome. However, the identification of non-steroidal therapies is necessary for long-term management toward reducing the steroid dose and side effects. The steroid-sparing treatments that have been reported to have some success are methotrexate, mycophenolate, azathioprine, cyclophosphamide, and cyclosporine (2–4). Targeted agents, including anti-IL-1 (anakinra and canakinumab), anti-IL-6 (tocilizumab), anti-tumor necrosis factor α (TNF-α) (adalimumab, infliximab, and etanercept), and Janus kinase inhibitors, have been proposed as possible treatments for VEXAS syndrome (2–4). In fact, high serum levels of IL-6 have been observed in patients with VEXAS syndrome (1, 6). The effectiveness of tocilizumab for other inflammatory conditions with high levels of IL-6, such as adult-onset Still’s disease, may suggest a role for this agent in the treatment of VEXAS syndrome as well (6). The present case also showed a systemic corticosteroid and an anti-IL-6 agent to have a significant and rapid effect. Kunishita et al. reported that the combination of tocilizumab and glucocorticoids allows the patients to continue treatment for at least 1 year without significant disease progression. Glucocorticoids are able to be reduced from the start of tocilizumab (7). Bourbon et al. noted that most treatments were only transiently effective; the median time to the next treatment was 3.4 months for adalimumab, 3.9 months for corticosteroids, 7.4 months for methotrexate, and 8 months for tocilizumab (8). This patient may also relapse and should be followed up carefully. It is well known that significant side effects of tocilizumab may include the reactivation of tuberculosis; infection by opportunistic bacteria, fungi, and viruses; and intestinal perforation. Of note, there have been two reports of intestinal perforation in patients with VEXAS syndrome receiving tocilizumab (5, 6). Certainly, additional data and long-term follow-ups are needed to further validate these findings.

Obiorah et al. said, VEXAS syndrome patient’s BM aspirate smears showed marked cytoplasmic vacuolization in hematopoietic precursors, including blasts and erythroid and myeloid precursors. Of note, vacuoles were predominantly found in early precursors (blasts, promyelocytes, and pronormoblasts). Vacuoles were also identified in eosinophils, monocytes, plasma cells, and megakaryocytes to a lesser degree. Lymphocytes were devoid of vacuoles (9). In this case we could confirm vacuolization in proerythroblast, promyelocyte, myelocyte, metamyelocyte. We could not confirm vacuolization in plasma cells which belong to the B cell lineage.

In previous reports of VEXAS syndrome, the *UBA1* variant was found only in neutrophilic dermatitis by skin biopsy, and not in leukocytoclastic vasculitis or septal panniculitis (10–14). This could lead to a distinction between clonal and paraclonal cutaneous involvements in VEXAS syndrome, which could in turn improve therapeutic outcomes (10). Zakine et al. reported that the infiltrates were perivascular and consisted of mature neutrophils with leukocytoclasia admixed with myeloperoxidase-positive CD163-positive myeloid cells with indented nuclei and lymphoid cells in eight patients with neutrophilic dermatosis (14). Sequencing analyses of bone marrow samples and skin lesion biopsies identified the same loss-of-function *UBA1* variant in all patients (14). VEXAS syndrome is caused by *UBA1* variants in myeloid lineage cells, including neutrophils and macrophages. CD68-positive M1 macrophages and CD163-positive M2 macrophages have been reported (14–16). M1-macrophages differentiate from inflammatory monocytes with TNF-α and interferon-γ, and are involved in controlling infections by pathogens and parasites. In contrast, M2 macrophages differentiate from tissue-resident monocytes with Th2 cytokines such as IL-4 and IL-13, and are involved in tissue repair. M2 macrophage polarization is closely related to Th2 immunity (17). In this case, M2 macrophages might be mainly involved in the pathogenesis based on histological MPO-CD163-positive cell infiltrates and elevated serum levels of TARC and IgE. The patient had a history of surgery for pulmonary aspergillosis. He had no history of bronchial asthma, but it is possible that he was allergic and had elevated IgE, as in allergic bronchopulmonary aspergillosis.

Neutrophils generate ROS and are involved in various functions, including cell signaling homeostasis, and carcinogenesis. We evaluated the oxidative stress levels by measuring the d-ROMs. d-ROM quantification is a simple method for detecting hydroperoxide levels, and clinical trials have shown the d-ROM test to be useful for evaluating oxidative stress (18). d-ROM levels were decreased after treatment with oral prednisone (15 mg/d) and the first injections of tocilizumab (400 mg). ROS are commonly associated with neutrophil extracellular trap (NET) formation. It is possible that d-ROM levels were linked to changes in NET formation in the patient. Of course, it is difficult to conclude that d-ROM is an indicator of disease activity in VEXAS syndrome based on only this one case. The AutoInflammatory Disease Alliance (AIDA) keeps an international registry that includes VEXAS syndrome cases (19). We hope that further studies will be planned.

The diagnostic work-up for a patient with suspected VEXAS syndrome should include the characteristic cutaneous lesions (e.g., neutrophilic dermatitis, chondritis, vasculitis) and investigations of inflammatory markers (e.g., CRP, serum ferritin), a bone marrow biopsy, pulmonary imaging, pulmonary function tests, and the necessary investigations to rule out differential diagnoses. However, VEXAS syndrome can be confirmed only by the presence of a somatic *UBA1* variant, usually in peripheral blood. The dermatologist is often the first clinician to suspect a diagnosis of VEXAS syndrome. Early recognition and diagnosis may lead to a better prognosis and targeted treatments in the near future.

## Data availability statement

The original contributions presented in this study are included in the article/supplementary material, further inquiries can be directed to the corresponding author.

## Ethics statement

Written informed consent was obtained from the individuals for the publication of any potentially identifiable images or data included in this article.

## Author contributions

HI and HO involved with the conception of the work and participated in the revision of the manuscript. NT, CT, YS, AKaw, YMw, and HO contributed to the data acquisition. NT, CT, HI, HO, HS, KM, and JH contributed to the data analysis and interpretation. NT and CT drafted the manuscript. All authors approved the submitted version.

## References

[1] Beck D Ferrada M Sikora K Ombrello A Collins J Pei W Somatic mutations in UBA1 and severe adult-onset autoinflammatory disease. *N Engl J Med.* (2020) 383:2628–38. 10.1056/NEJMoa2026834 33108101 PMC7847551

[2] Stubbins R Cherniawsky H Chen L Nevill T. Innovations in genomics for undiagnosed diseases: vacuoles, E1 enzyme, X-linked, autoinflammatory, somatic (VEXAS) syndrome. *CMAJ.* (2022) 194:E524–7. 10.1503/cmaj.211770 35410861 PMC9001005

[3] Sterling D Duncan M Philippidou M Salisbury J Kulasekararaj A Basu T. AS syndrome (vacuoles, E1 enzyme, X-linked, autoinflammatory, somatic) for the dermatologist. *J Am Acad Dermatol.* (2022). [Epub ahead of print]. 10.1016/j.jaad.2022.01.042 35121074

[4] Georgin-Lavialle S Terrier B Guedon A Heiblig M Comont T Lazaro E Further characterization of clinical and laboratory features in VEXAS syndrome: large-scale analysis of a multicentre case series of 116 French patients. *Br J Dermatol.* (2022) 186:564–74. 10.1111/bjd.20805 34632574

[5] Thomas V Penmetcha M. Myelodysplastic syndrome associated with auto-immune inflammatory disease in VEXAS syndrome. *J Hematol.* (2021) 10:274–6. 10.14740/jh940 35059089 PMC8734488

[6] Kirino Y Takase-Minegishi K Tsuchida N Hirahara L Kunishita Y Yoshimi R Tocilizumab in VEXAS relapsing polychondritis: a single-center pilot study in Japan. *Ann Rheum Dis.* (2021) 80:1501–2. 10.1136/annrheumdis-2021-220876 34260381

[7] Kunishita Y Kirino Y Tsuchida N Maeda A Sato Y Takase-Minegishi K Case report: tocilizumab treatment for VEXAS syndrome with relapsing polychondritis: a single-center, 1-year longitudinal observational study in Japan. *Front Immunol.* (2022) 13:901063. 10.3389/fimmu.2022.901063 35769485 PMC9234115

[8] Bourbon E Heiblig M Gerfaud Valentin M Barba T Durel C Lega J Therapeutic options in VEXAS syndrome: insights from a retrospective series. *Blood.* (2021) 137:3682–4. 10.1182/blood.2020010177 33619558

[9] Obiorah I Patel B Groarke E Wang W Trick M Ombrello A Benign and malignant hematologic manifestations in patients with VEXAS syndrome due to somatic mutations in UBA1. *Blood Adv.* (2021) 5:3203–15. 10.1182/bloodadvances.2021004976 34427584 PMC8405186

[10] Staels F Betrains A Woei-A-Jin F Boeckx N Beckers M Bervoets A Case report: VEXAS syndrome: from mild symptoms to life-threatening macrophage activation syndrome. *Front Immunol.* (2021) 12:678927. 10.3389/fimmu.2021.678927 34046042 PMC8147557

[11] Lacombe V Beucher A Urbanski G Le Corre Y Cottin L Croué A Distinction between clonal and paraclonal cutaneous involvements in VEXAS syndrome. *Exp Hematol Oncol.* (2022) 16:6. 10.1186/s40164-022-00262-5 35172893 PMC8848791

[12] Afsahi V Christensen R Alam M. VEXAS syndrome in dermatology. *Arch Dermatol Res.* (2022). [Epub ahead of print]. 10.1007/s00403-022-02340-4 35201420

[13] Sterling D Duncan M Philippidou M Salisbury J Kulasekararaj A Basu T. VEXAS syndrome (vacuoles, E1 enzyme, X-linked, autoinflammatory, somatic) for the dermatologist. *J Am Acad Dermatol.* (2022). [Epub ahead of print]. 10.1016/j.jaad.2022.01.042 35121074

[14] Zakine E Schell B Battistella M Vignon-Pennamen M Chasset F Mahévas T UBA1 variations in neutrophilic dermatosis skin lesions of patients with VEXAS syndrome. *JAMA Dermatol.* (2021) 157:1349–54. 10.1001/jamadermatol.2021.3344 34495287 PMC8427502

[15] Sloan B. This month in JAAD case reports: November 2021. vacuoles, E1 enzyme, X-linked, autoinflammatory, somatic (VEXAS) syndrome-a newly described autoinflammatory disease. *J Am Acad Dermatol.* (2021) 85:1111. 10.1016/j.jaad.2021.09.006 34509541

[16] Ciprian G. Adverse reaction to COVID-19 mRNA vaccination in a patient with VEXAS syndrome. *Cureus.* (2022) 14:e23456. 10.7759/cureus.23456 35481304 PMC9034849

[17] Wang N Liang H Zen K. Molecular mechanisms that influence the macrophage m1-m2 polarization balance. *Front Immunol.* (2014) 28:614. 10.3389/fimmu.2014.00614 25506346 PMC4246889

[18] Trotti R Carratelli M Barbieri M. Performance and clinical application of a new, fast method for the detection of hydroperoxides in serum. *Panminerva Med.* (2002) 44:37–40. 11887090

[19] Vitale A Caggiano V Della Casa F Hernández-Rodríguez J Frassi M Monti S Development and implementation of the AIDA international registry for patients with VEXAS syndrome. *Front Med.* (2022) 11:926500. 10.3389/fmed.2022.926500 35899212 PMC9309690

